# Comparison of cognitive performance in first-episode drug-naïve schizophrenia, bipolar II disorder, and major depressive disorder patients after treatment

**DOI:** 10.1186/s12888-024-05897-8

**Published:** 2024-06-11

**Authors:** Chaohua Tang, Wei Huang, Yukang Tan, Yiliang Liu, Guangen Zheng, Bin Li, Wensheng Chen, Yu Yang, Guohong Xu, Xiaoling Li, Caixia Xu, Guojun Xie, Jiaquan Liang

**Affiliations:** 1 Department of Psychiatry, The Third People’s Hospital of Foshan, Guangdong, People’s Republic of China; 2 Key Laboratory of Ethnomedicine of Ministry of Education, Center On Translational Neuroscience, School of Pharmacy, Minzu University of China, Beijing, China

**Keywords:** Schizophrenia, Bipolar II disorder, Major depressive disorder, Cognitive dysfunction

## Abstract

**Background:**

Cognitive impairment is a recognized fundamental deficit in individuals diagnosed with schizophrenia (SZ), bipolar II disorder (BD II), and major depressive disorder (MDD), among other psychiatric disorders. However, limited research has compared cognitive function among first-episode drug-naïve individuals with SZ, BD II, or MDD.

**Methods:**

This study aimed to address this gap by assessing the cognitive performance of 235 participants (40 healthy controls, 58 SZ patients, 72 BD II patients, and 65 MDD patients) using the Repeatable Battery for the Assessment of Neuropsychological Status (RBANS) before and after 12 weeks of treatment in SZ, BD II, and MDD patients. To clarify, the healthy controls only underwent RBANS testing at baseline, whereas the patient groups were assessed before and after treatment. The severity of symptoms in SZ patients was measured using the Positive and Negative Syndrome Scale (PANSS), and depression in BD II and MDD patients was assessed using the Hamilton Depression Scale-24 items (HAMD-24 items).

**Results:**

Two hundred participants completed the 12-week treatment period, with 35 participants dropping out due to various reasons. This group included 49 SZ patients, 58 BD II patients, and 53 MDD patients. Among SZ patients, significant improvements in immediate and delayed memory were observed after 12 weeks of treatment compared to their initial scores. Similarly, BD II patients showed significant improvement in immediate and delayed memory following treatment. However, there were no significant differences in RBANS scores for MDD patients after 12 weeks of treatment.

**Conclusions:**

In conclusion, the findings of this study suggest that individuals with BD II and SZ may share similar deficits in cognitive domains. It is important to note that standardized clinical treatment may have varying degrees of effectiveness in improving cognitive function in patients with BD II and SZ, which could potentially alleviate cognitive dysfunction.

## Introduction

Psychiatric disorders are a significant public health challenge, impacting almost 450 million people worldwide [1]. These disorders include schizophrenia (SZ), bipolar disorder (BD), major depressive disorder (MDD), and anxiety disorders, among others, which affect emotion, behavior, and cognition [2, 3]. Moreover, individuals with psychiatric disorders face a higher risk of premature mortality compared to the general population [4]. This underscores the substantial difficulties society faces in addressing the healthcare, emotional, and economic implications tied to premature mortality among those with psychiatric disorders.

SZ, BD, and MDD are severe psychiatric disorders with complex etiologies and pathophysiologies, often characterized by overlapping clinical symptoms. While current treatments successfully target symptoms like anxiety, delusions, or insomnia, uncovering the underlying causes and providing long-term relief remain significant challenges [5]. Unfortunately, the treatment of cognitive dysfunction, a core deficit in psychiatric disorders, has shown little to no improvement over time, and may have even worsened [6]. SZ patients commonly exhibit cognitive deficits in attention, processing speed, spatial memory, and executive function [7]. BD is a complex mental health condition characterized by episodes of extreme mood swings ranging from mania to depression [8]. BD is divided into two main types based on the types of mood episodes experienced: Bipolar I Disorder (BD-I), characterized by recurrent episodes of mania and depression.; Bipolar II Disorder (BD-II), characterized by episodes of major depression and hypomania, but not full-blown mania [9]. BD II and MDD patients may also experience cognitive dysfunction in attention, processing speed, verbal learning/memory, and executive function domains [10, 11]. Therefore, cognitive function can serve as a valuable intermediate endophenotype in identifying the underlying pathogenesis associated with shared symptoms among psychiatric patients [12].

Comparing cognitive functions across different psychiatric disorders can enhance our understanding of their distinct characteristics and cognitive profiles. While some studies have investigated cognitive functions in psychiatric disorders, others have focused on specific disorders [13, 14]. Study specifically comparing cognitive function among SZ, BD II, and MDD, particularly in first-episode drug-naïve patients with psychosis, remains scarce. However, methodological discrepancies and variations in assessment tools used across studies may limit the comparability and generalizability of findings regarding cognitive dysfunctions in psychiatric disorders.

Cognitive heterogeneity refers to the wide range of cognitive abilities and deficits observed among individuals with SZ or BD, even within the same diagnostic category. This variability can be particularly pronounced along the SZ-BD spectrum, where individuals may exhibit symptoms of both disorders or characteristics of related conditions [15]. Both SZ and BD are associated with cognitive deficits that can include impairments in attention, memory, executive functioning, and processing speed. However, the specific nature and severity of these deficits can vary widely among individuals [16]. Within SZ and BD, there are subtypes that may be associated with different cognitive profiles [17]. SZ is characterized by positive symptoms (e.g., delusions, speech disorders), negative symptoms (e.g., diminished emotional expression), and persistent cognitive dysfunction throughout the course of the illness [18, 19]. Considering its close association with functional outcomes, cognitive dysfunction holds paramount importance. Cognition encompasses attention, cognitive control, problem-solving, processing speed, social cognition, visual learning, verbal learning, and working memory [20]. Impairments in memory, attention, problem-solving, and processing speed are especially pronounced in SZ [21]. BD II is linked to cognitive impairment across multiple functional domains, persisting even after clinical rehabilitation [22, 23]. The most affected domains include attention, language learning and memory, and executive function, while premorbid intelligence remains preserved [24, 25]. Although cognitive dysfunctions are present throughout all illness phases, they are more pronounced during acute episodes in euthymic BD II patients [8]. Furthermore, cognitive impairment in BD II patients has been reported to be similar to, albeit less severe than, that observed in SZ patients [26].

It is crucial for the continuous spectrum of illness in SZ and BD. It has been confirmed that cognitive deficits may not be discrete entities but rather part of a continuum that spans across different psychiatric disorders [27]. Additionally, it has found overlapping cognitive profiles between these two disorders, particularly in domains such as working memory, processing speed, and executive functions [28]. Discussing cognitive dysfunction in the context of a continuous spectrum of illness can provide valuable insights into the shared pathophysiological mechanisms that may contribute to cognitive impairments in schizophrenia and bipolar illness. Researchers and clinicians may be better equipped to develop more effective cognitive-based interventions that target common deficits across SZ, BD, and MDD by acknowledging the overlap in cognitive profiles. This could lead to improved outcomes for individuals with these conditions, as well as those with other psychiatric disorders that share similar cognitive impairments.

There is a number of studies available that compares cognitive performance after treatment [29, 30], it is a crucial aspect of understanding the effectiveness of the treatment. Comparing pre- and post-treatment cognitive performance allows us to assess the impact of the treatment on cognitive function and gain insights into its potential benefits. While individual studies may focus on the specific effects of treatment on cognitive performance [31], a comprehensive understanding of the changes in cognitive abilities before and after treatment is essential. Therefore, in this study, we aim to fill this gap by comparing cognitive performance before and after treatment to provide valuable insights into the effectiveness of the treatment on cognitive function.

This study was conducted to assess the extent of cognitive dysfunction in first-episode drug-naïve patients with SZ, BD II, and MDD after 12 weeks of follow-up treatment. Initially, we evaluated the Positive and Negative Syndrome Scale (PANSS), Hamilton Depression Scale-24 items (HAMD-24 items), and Repeatable Battery for the Assessment of Neuropsychological Status (RBANS) as baseline predictors. Subsequently, we compared RBANS scores and examined the differences in cognitive functions among SZ, BD II, and MDD during the 12-week post-treatment follow-up.

## Method

### Participants

Participants with psychosis were recruited from the Third People’s Hospital of Foshan, China. The patients' diagnoses were administered by a trained professional who is qualified in neuropsychological assessments and were confirmed according to the Diagnostic and Statistical Manual of Mental Disorders-5 (DSM-5). Relevant clinical information was obtained through the Structured Clinical Interview Version of the DSM-5(R) Clinical Edition (SCID-5-CV). Healthy control (HC) participants were recruited from the local community in Foshan through advertisements. HC participants were required to have no familial history of psychosis.

### Procedure

This study obtained approval from the Medical Ethics Committee of the Third People’s Hospital of Foshan, and all participants provided informed written consent. The PANSS is a widely used rating scale for assessing the severity of symptoms in patients diagnosed with SZ. It is designed to measure positive symptoms, negative symptoms, and general psychopathology [32]. The RBANS is a neuropsychological assessment tool used to evaluate cognitive function across various domains, including attention, memory, language, and executive functioning [33]. It was administered to individuals who is qualified in neuropsychological assessments.

Our approach to treatment involves pharmacotherapy, focusing exclusively on medication-based interventions tailored to address the patient's mental health needs. We prioritize a personalized approach to psychiatric treatment, excluding non-pharmacological methods such as psychotherapy, transcranial magnetic stimulation (TMS), and psychedelic therapy. The participants underwent a baseline assessment battery, with follow-up assessments conducted at 12 weeks. RBANS scores were assessed at baseline and at the 12-week mark for all participants. Additionally, PANSS scores were evaluated for SZ patients, and HAMD scores were evaluated for BD II and MDD patients. The treatment choices were tailored to each individual's specific condition.

### Inclusion and exclusion criteria

The inclusion criteria comprised of (1) recent first-episode drug-naïve psychotic illness, (2) patients without a familial history or underlying diseases, and (3) a diagnosis of SZ, BD II, or MDD according to the DSM-5. Exclusion criteria included other mental disorders, a familial history of mental disorders, brain organic diseases, physical diseases, substance abuse (drugs, alcohol), traumatic brain injuries, neurological diseases, excessive use of sleep drugs.

### Outcome

To clarify, the HCs only underwent RBANS testing at baseline, whereas the patient groups were assessed before and after treatment. HAMD is a questionnaire utilized to assess depression severity and remission. Scores between 8 and 20 indicate mild depression, scores between 20 and 35 indicate moderate depression, and scores above 35 indicate severe depression. Scores below 8 indicate full remission. PANSS scores were used to evaluate the remission and severity of SZ (positive and negative symptoms). RBANS scores were measured at baseline and at the end of the study to assess the cognitive function of the patients.

### Statistical Analysis

Descriptive statistics were calculated to characterize the sample at baseline. One-way ANOVA was employed to compare the four groups. To assess the effectiveness of treatment for SZ, BD II, and MDD, two-way ANOVA was performed to evaluate the changes in PANSS and HAMD scores between baseline and the end of the study. Similarly, two-way ANOVA was utilized to compare RBANS scores at baseline and the end of the study. Pearson's correlation coefficients were calculated to quantify the linear relationship between improvements in cognitive function and clinical symptoms. A p-value of < 0.05 was considered statistically significant, and the data are presented as mean ± standard deviation (SD). All statistical analyses were performed using SPSS Statistics version 21.0 for Windows (IBM Corporation, Armonk, USA).

## Results

### Patient Characteristics

**Fig. 1:**
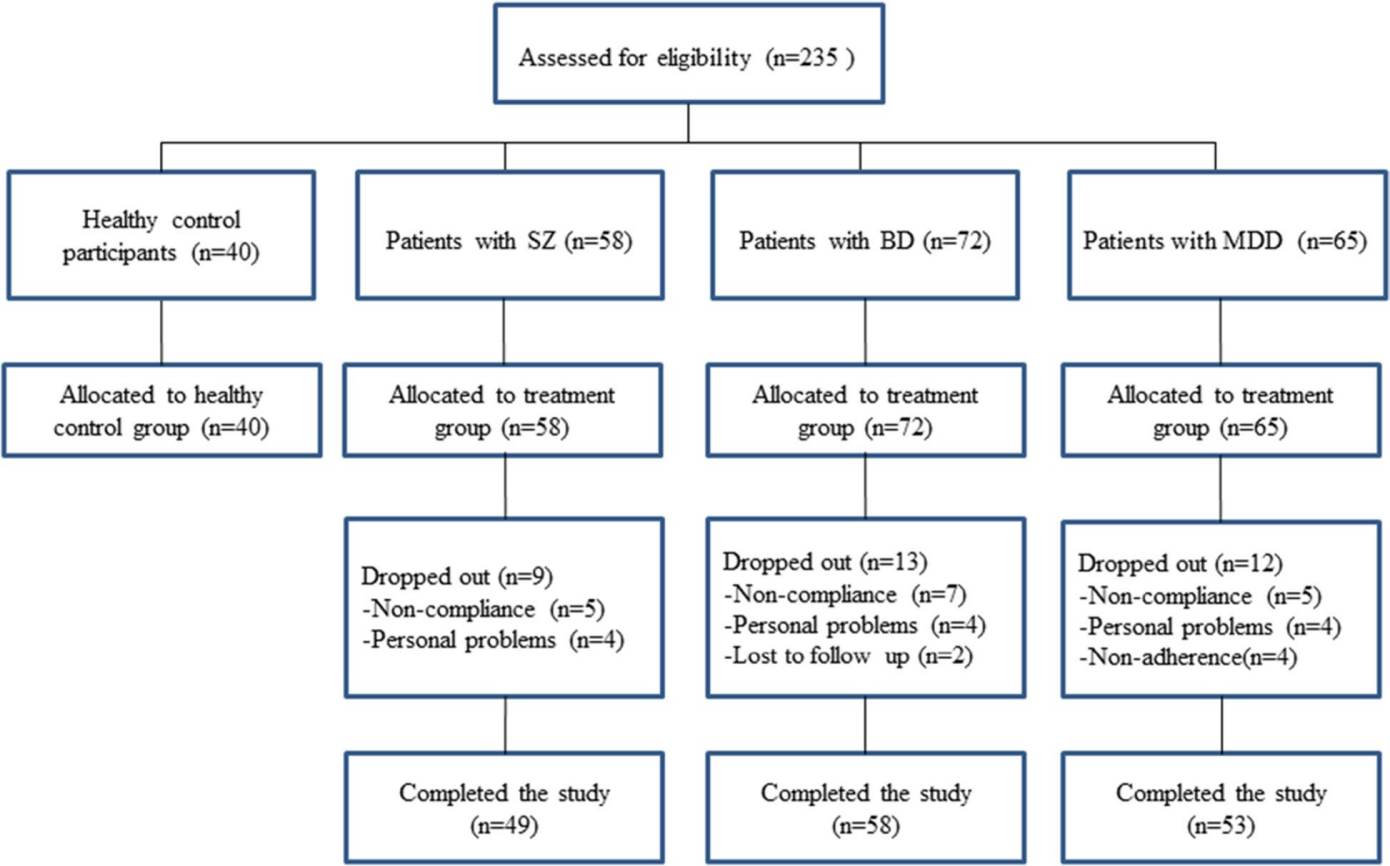
Flow diagram illustrating the inclusion and exclusion of patients in the study

A total of 235 participants underwent initial screening, including 40 HCs and patients with SZ, BD II, and MDD (*n* = 58, *n* = 72, and *n* = 65, respectively). Unfortunately, 35 participants dropped out of the study for various reasons. Ultimately, 200 participants (n_HC_ = 40, n_SZ_ = 49, n_BD II_ = 58, n_MDD_ = 53) completed the study (Fig. 1).

The demographic and clinical characteristics of the participants are presented in Table 1. There were no significant differences in age, gender, or education level among the groups. The SZ group exhibited significantly higher PANSS scores compared to the HC group (*P* < 0.05). In the BD II and MDD groups, baseline HAMD scores were significantly higher than those in the HC group (*P* < 0.05). Furthermore, baseline RBANS scores in the SZ group were significantly lower than those in the HC group. In the BD II group, except for visuospatial construction and language, all other cognitive scores were lower compared to the HC group. No significant differences in cognitive functioning were observed between the MDD and HC groups. Additionally, there were no significant differences in baseline characteristics between participants who completed the study and those who dropped out (Table 2).

**Table 1:** Baseline characteristics of the participants

	Healthy controls	Schizophrenia (P-Value)	BD II (P-Value)	MDD	F/t	*P* value
Participants	40	49	58	53		
Age (years)	37.35 ± 13.13	39 ± 8.86	36.64 ± 10.08	36.6 ± 9.96	0.137	*P* = 0.365
Gender (M/F)	16/24	21/28	24/34	20/33	51.790	*P* = 0.769
Education						
Less than 9 years, n (%)	14 (35%)	17 (34.7%)	18 (31%)	13 (25.2%)	0.154	*P* = 0.564
9 years, n (%)	6 (15%)	9 (18.4%)	11 (19.0%)	12 (22.6%)		
10–12 years, n (%)	6 (15%)	5 (10.2%)	9 (15.5%)	8 (15.1%)		
13–17 years, n (%)	14 (35%)	18 (36.7%)	20 (34.5%)	19 (35.8%)		
Baseline HAMD score	2.88 ± 4.36		16.75 ± 8.41^*^	24.89 ± 7.64^*^	105.3	*P* < 0.001
Baseline PANSS score	30.05 ± 0.32	65 ± 19.48^*^ (*P* = 0.015)			15.33	*P* < 0.001
RBANS	176.50 ± 36.50	130.65 ± 33.37^*^ (*P* < 0.001)	160.29 ± 31.05^*^ (*P* = 0.020)	174.57 ± 32.05	284.4	*P* < 0.001
Immediate memory (Learning)	28.15 ± 7.09	18.37 ± 6.58^*^ (*P* < 0.001)	22.17 ± 5.91^*^ (*P* < 0.001)	26.04 ± 6.46	20.76	*P* < 0.001
Immediate memory (Story Memory)	14.63 ± 5.82	6.45 ± 4.71^*^ (*P* < 0.001)	9.69 ± 4.46^*^ (*P* < 0.001)	13.36 ± 5.63	24.48	*P* < 0.001
Visuospatial Construction	17.68 ± 2.53	17.16 ± 2.62	17.43 ± 3.17	17.72 ± 3.18	21.76	*P* < 0.001
Language	18.8 ± 4.34	12.35 ± 4.80^*^ (*P* < 0.001)	17.19 ± 4.44	17.58 ± 4.39	18.87	*P* < 0.001
Attention (Digit span)	14.23 ± 2.21	11.12 ± 2.49^*^ (*P* < 0.001)	13.03 ± 2.44^*^ (*P* = 0.015)	13.53 ± 2.74	13.30	*P* < 0.001
Attention (Coding)	48.95 ± 14.93	30.78 ± 11.98^*^ (*P* < 0.001)	41.26 ± 12.96^*^ (*P* = 0.008)	46.60 ± 13.20	17.67	*P* < 0.001
Delayed memory (List Recall)	6.55 ± 3.19	3.35 ± 2.60^*^ (*P* < 0.001)	4.67 ± 2.38^*^ (*P* = 0.001)	5.75 ± 2.96	11.59	*P* < 0.001
Delayed memory (List Recognition)	20.20 ± 4.96	17.63 ± 2.67^*^ (*P* = 0.003)	18.84 ± 1.66^*^ (*P* = 0.045)	19.40 ± 1.15	6.849	*P* < 0.001
Delayed memory (Story Recall)	7.33 ± 3.87	3.14 ± 2.82^*^ (*P* < 0.001)	4.74 ± 2.66^*^ (*P* < 0.001)	7.09 ± 3.77	17.85	*P* < 0.001
Delayed memory (Figure Recall)	14.10 ± 4.71	10.20 ± 5.59^*^ (*P* < 0.001)	11.26 ± 4.67^*^ (*P* = 0.004)	13.57 ± 4.13	7.174	*P* < 0.001

*BD II* Bipolar II disorder, *MDD* Major depression disorder, *HAMD* Hamilton depression scale, *HAMA* Hamilton anxiety scale, *RBANS* Repeatable battery for the assessment of neuropsychological status. ^*^P < 0.05 compared with healthy control group respectively; values are expressed as mean ± standard deviation

**Table 2:** Baseline characteristics of the completed and dropped out of treatment

	Schizophrenia		BD II		MDD	
	completed	dropped out	completed	dropped out	completed	dropped out
Participants	49	9	58	13	53	12
Age (years)	39 ± 8.86	35.33 ± 5.64	36.64 ± 10.08	36.46 ± 7.32	36.6 ± 9.96	36.33 ± 7.37
Gender (M/F)	21/28	4/5	24/34	5/8	20/33	5/7
Education						
Below 9 years, n (%)	17 (34.7%)	3(33.3%)	18 (31%)	5(38.5%)	13 (25.2%)	4(33.3%)
Up to 9 years, n (%)	9 (18.4%)	2(22.2%)	11 (19.0%)	2(15.4%)	12 (22.6%)	2(11.1%)
10–12 years, n (%)	5 (10.2%)	1(11.1%)	9 (15.5%)	2(15.4%)	8 (15.1%)	2(11.1%)
13–17 years, n (%)	18 (36.7%)	3(33.3%)	20 (34.5%)	4(30.8%)	19 (35.8%)	4(33.3%)
Baseline HAMD score			16.75 ± 8.41	14.31 ± 4.63	24.89 ± 7.64	26.25 ± 5.59
Baseline PANSS score	65 ± 19.48	67.33 ± 20.13				
RBANS						
	130.65 ± 33.37	129.3 ± 20.41	160.29 ± 31.05	155.3 ± 16.68	174.57 ± 32.05	166.8 ± 18.01
Immediate memory (Learning)	18.37 ± 6.58	18.67 ± 3.90	22.17 ± 5.91	23.38 ± 4.11	26.04 ± 6.46	26.33 ± 3.31
Immediate memory (Story Memory)	6.45 ± 4.71	5.44 ± 3.24	9.69 ± 4.46	8.85 ± 3.02	13.36 ± 5.63	14.46 ± 3.60
Visuospatial Construction	17.16 ± 2.62	17.89 ± 1.27	17.43 ± 3.17	17.23 ± 1.48	17.72 ± 3.18	18.58 ± 1.44
Language	12.35 ± 4.80	10.89 ± 1.54	17.19 ± 4.44	18.08 ± 2.72	17.58 ± 4.39	16.83 ± 3.26
Attention (Digit span)	11.12 ± 2.49	11.44 ± 1.33	13.03 ± 2.44	12.23 ± 1.54	13.53 ± 2.74	13.75 ± 2.00
Attention (Coding)	30.78 ± 11.98	32.0 ± 4.09	41.26 ± 12.96	38.12 ± 5.00	46.60 ± 13.20	45.23 ± 5.54
Delayed memory (List Recall)	3.35 ± 2.60	3.22 ± 1.39	4.67 ± 2.38	4.69 ± 1.93	5.75 ± 2.96	6.67 ± 1.76
Delayed memory (List Recognition)	17.63 ± 2.67	16.67 ± 1.50	18.84 ± 1.66	17.62 ± 1.61	19.40 ± 1.15	18.17 ± 1.03
Delayed memory (Story Recall)	3.14 ± 2.82	4.02 ± 1.64	4.74 ± 2.66	4.16 ± 1.73	7.09 ± 3.77	7.17 ± 2.69
Delayed memory (Figure Recall)	10.20 ± 5.59	11.33 ± 5.07	11.26 ± 4.67	11.46 ± 3.46	13.57 ± 4.13	14.33 ± 3.26

*BD II* Bipolar II disorder, *MDD* Major depression disorder, *HAMD* Hamilton depression scale, *HAMA* Hamilton anxiety scale, *RBANS* Repeatable battery for the assessment of neuropsychological status

### Outcomes

**Fig. 2:**
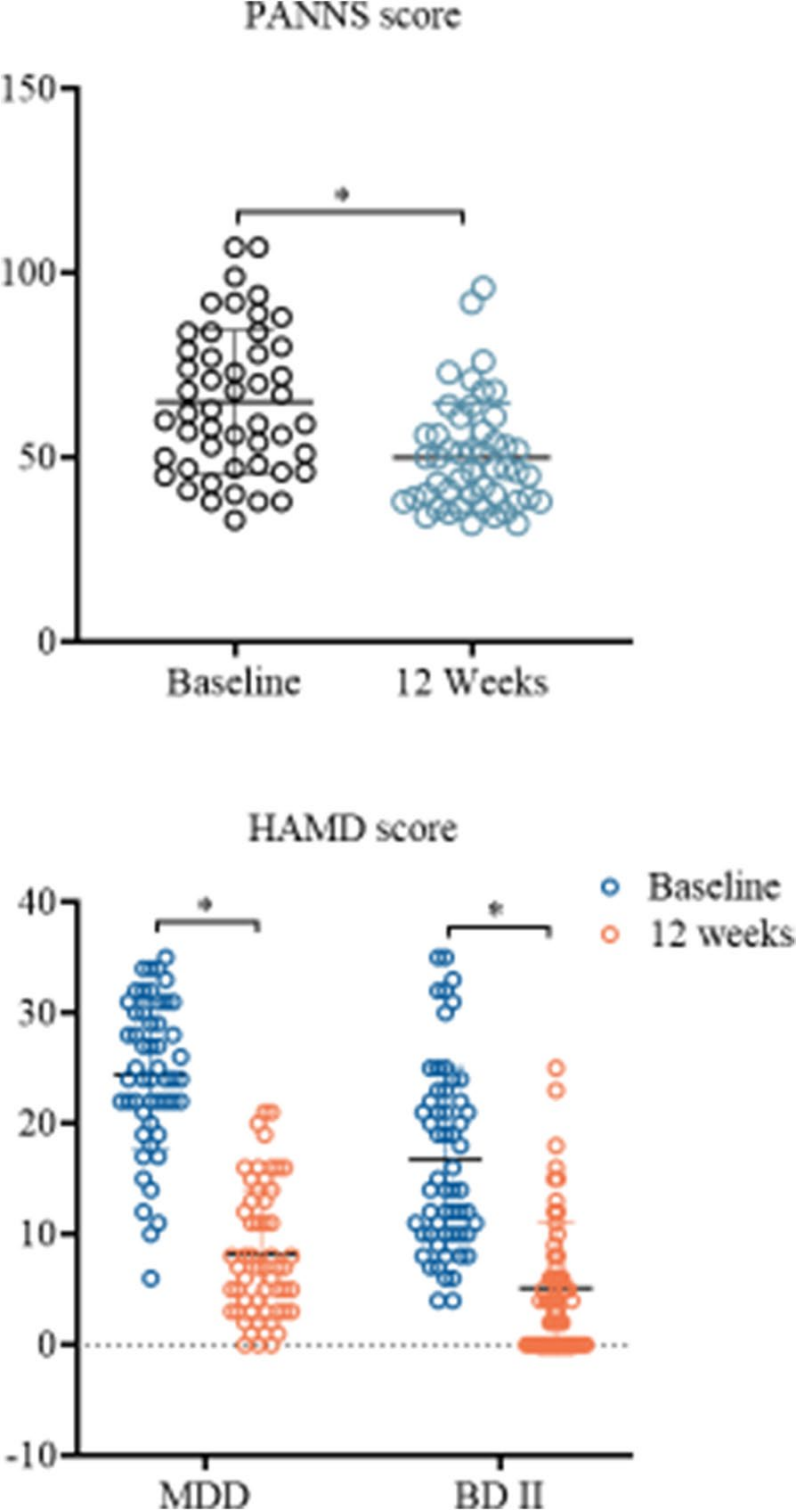
Comparison of PANSS and HAMD scores between baseline and the 12th week of treatment. PANSS, Positive and Negative Syndrome Scale; HAMD, Hamilton Depression Scale

**Fig. 3:**
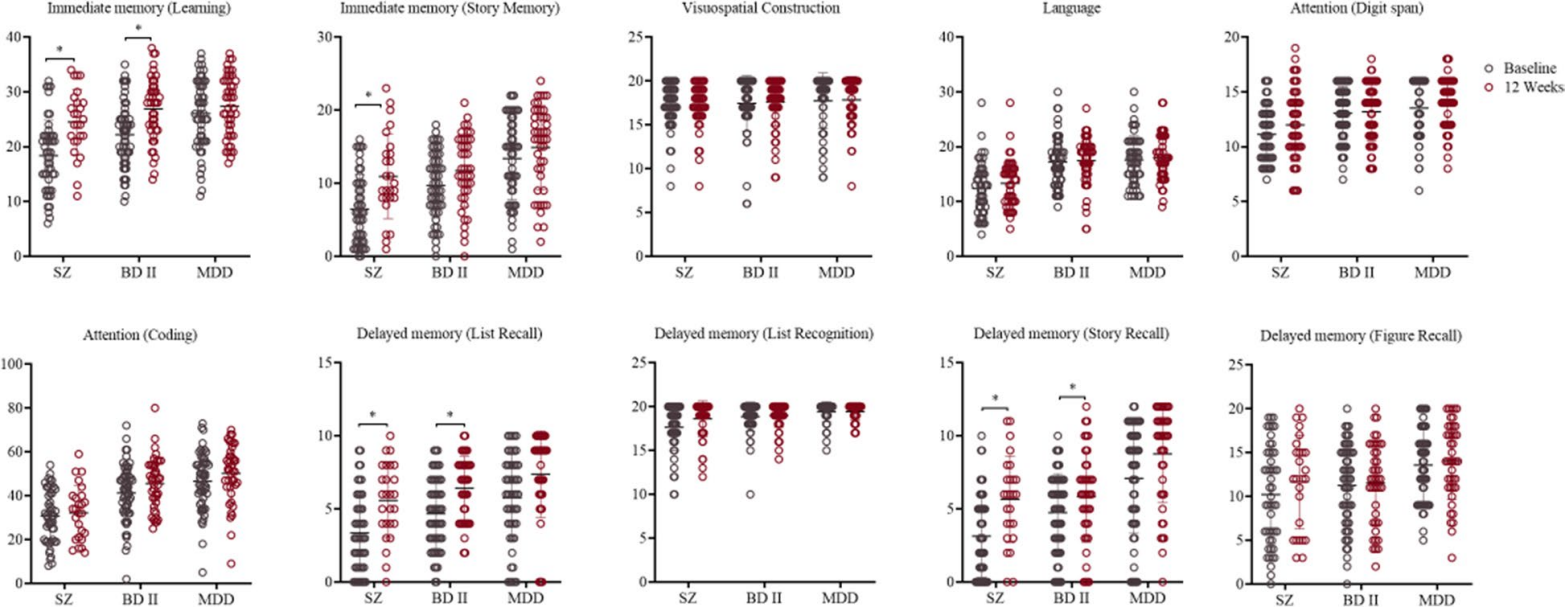
Comparison of RBANS scores between baseline and the 12th week of treatment for SZ, BD II, and MDD patients. SZ, schizophrenia; BD II, bipolar II disorder; MDD, major depressive disorder; RBANS, Repeatable Battery for the Assessment of Neuropsychological Status

Significant improvements were observed in HAMD scores for patients with BD II and MDD at the 12-week follow-up after treatment. Similarly, patients with SZ showed a significant reduction in PANSS scores (Fig. 2). Cognitive function was assessed using RBANS, including measures of immediate memory, visuospatial construction, language, attention, and delayed memory. Results indicated that patients with SZ demonstrated substantial improvements in both immediate memory (learning and story memory) and delayed memory (story recall) after 12 weeks of treatment compared to their initial scores. Similarly, BD II patients showed significant improvement in both immediate memory (learning) and delayed memory (story recall) following treatment. However, no significant differences in RBANS scores were observed after 12 weeks of treatment compared to baseline for patients with MDD (Fig. 3).

### Pearson correlation analysis

**Fig. 4:**
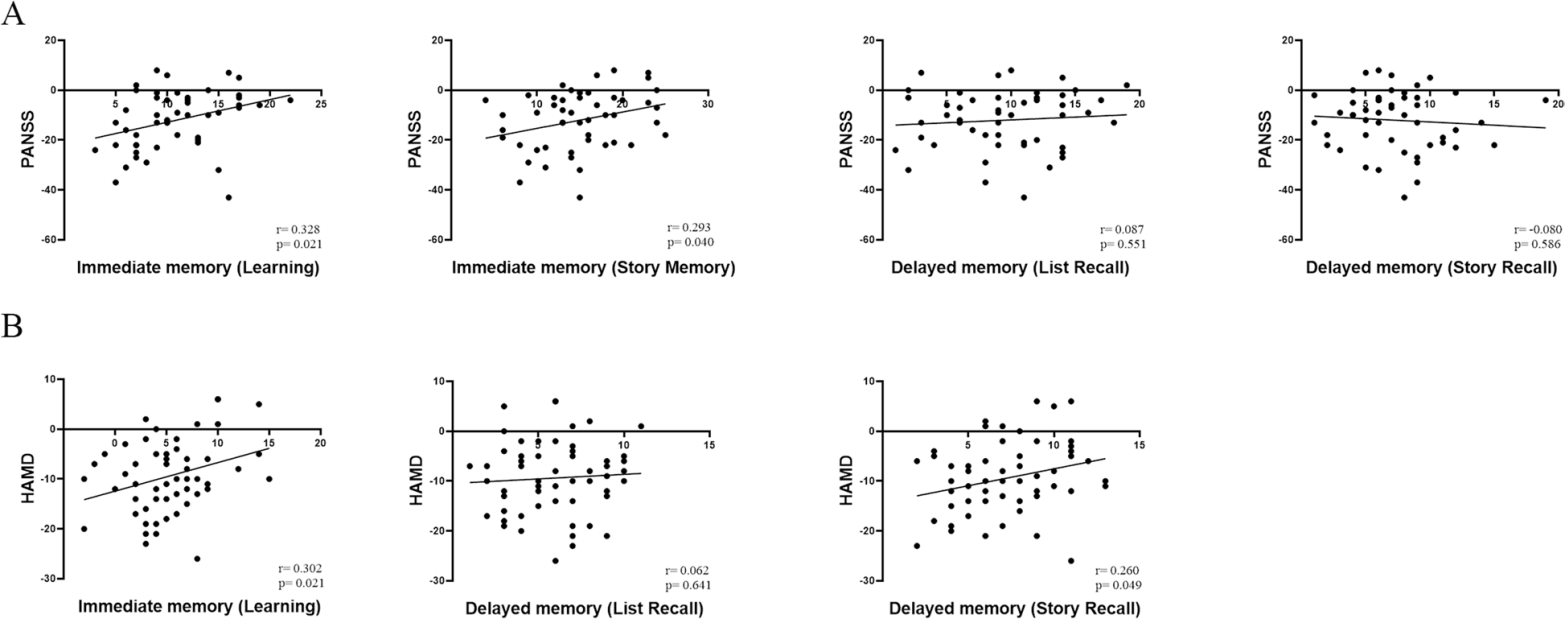
Pearson correlation analyses of **A** improvement values of the PANSS with the immediate memory (learning and story memory), delayed memory (list recall and story recall) score; and **B** the improvement values of HAMD with immediate memory (learning and story memory) score and delayed memory (story recall) score. PANSS, Positive and Negative Syndrome Scale; HAMD, Hamilton Depression Scale

Pearson correlation analysis revealed a positive correlation between improvement in PANSS scores and immediate memory scores (learning and story memory) (r_learning = 0.328, *p* = 0.021; r_story memory = 0.293, *p* = 0.040). Similarly, improvement in HAMD scores positively correlated with immediate memory scores (learning) (*r* = 0.302, *p* = 0.021) and delayed memory scores (story recall) (*r* = 0.260, *p* = 0.049) (Fig. 4).

## Discussion

The SZ group displayed significantly higher PANSS scores compared to the HC group, indicating greater symptom severity. Additionally, the BD II and MDD groups had significantly higher HAMD scores at baseline compared to the HC group, suggesting higher levels of depressive symptoms. Moreover, baseline scores on the RBANS were significantly lower in the SZ group compared to the HC group, indicating reduced cognitive function. In the BD II group, except for visuospatial construction and language, lower cognitive scores were observed in various domains compared to the HC group. The treatment outcomes revealed significant improvements in HAMD scores for patients with BD II and MDD after the 12-week follow-up, suggesting the effectiveness of the treatment in reducing depressive symptoms in these groups. Similarly, patients with SZ showed a significant reduction in PANSS scores, indicating a reduction in overall symptom severity for this group. These findings confirm the therapeutic effects of the interventions on clinical symptoms in patients with BD II, MDD, and SZ. Furthermore, the assessment of cognitive function using RBANS demonstrated promising results. Patients with SZ exhibited substantial improvements in both immediate and delayed memory after 12 weeks of treatment compared to their initial scores. Similarly, BD II patients showed significant improvement in both immediate and delayed memory following treatment. These findings suggest that the interventions had a positive impact on cognitive function in patients with SZ and BD II. However, no significant differences in RBANS scores were observed for patients with MDD after 12 weeks of treatment compared to baseline.

It has been consistently observed that both SZ and BD II characterized by cognitive dysfunction [34]. In our study, we found that both SZ and BD II patient groups consistently exhibited inferior performance compared to the HC group on cognitive tests at baseline and after 12 weeks of treatment. At the end of the study, SZ patients demonstrated significant differences in learning, story memory, and story recall. Similarly, the BD II patient group showed notable differences in learning and story recall. While the SZ and BD II groups displayed improved performance in delayed memory tasks (list recall, figure recall), these results did not reach statistical significance. It is worth noting that there were no significant improvements observed in visuospatial construction, language, or attention in any of the patient groups. Temporal processing deficits have been reported in individuals with SZ and BD II, and it is believed that these functional impairments underlie the observed deficits in aspects related to planning and decision-making [35, 36]. Thus, substantial improvements were observed in learning and memory, where significant impairment was present initially, after the treatment. The absence of significant differences in visuospatial construction, language, and attention may be attributed to task difficulty and the psychometric properties of the cognitive tests.

In our study, we observed that SZ patients exhibited lower RBANS scores at baseline compared to the HC group, consistent with previous findings regarding cognitive changes in SZ patients at the time of diagnosis [37]. Effective cognitive interventions have the potential to alleviate the limitations imposed by cognitive impairment, enabling patients to fully utilize their community functioning abilities, which are currently underutilized [38]. Our study focused on comparing the cognitive function of recently diagnosed drug-naïve SZ patients at the 12-week mark of psychiatric treatment, assessing performance at baseline and the end of the study. Notably, we observed significant improvements in learning, story memory, and story recall for SZ patients after 12 weeks of treatment. To ensure the comparability of cognitive dysfunction, we specifically selected recently diagnosed drug-naïve SZ patients to exclude the influence of confounding factors.

BD II is characterized by cognitive impairment across various functional domains, which often persists even after clinical rehabilitation [22, 23]. The most affected domains include attention, language learning and memory, and executive function, while premorbid intelligence appears to be preserved [24, 25]. Although cognitive dysfunction is present throughout all phases of BD II, it tends to be more pronounced during acute episodes [8]. Previous studies have reported that cognitive impairment in BD II is similar to, albeit less severe than, that observed in SZ patients [26]. In our study, we observed cognitive decline in recently diagnosed drug-naïve BD II patients compared to HCs. Moreover, the cognitive ability of BD II patients was found to be superior to that of SZ patients. Similar to the treatment approach for SZ, many studies assessing pharmacological therapy for cognitive dysfunction often overlook illness severity, past treatments, and comorbid symptoms. It remains unclear to what extent the observed cognitive dysfunctions in BD II are attributed to subsyndromal symptoms rather than the disorder itself or its treatment [39, 40]. Our findings indicate that recently diagnosed drug-naïve BD II patients showed improvements in cognitive functions, including learning and story recall, after 12 weeks of treatment. As for MDD patients, their baseline RBANS scores were lower compared to HCs, consistent with previous research30. Cognitive functions also improved in the MDD group after the 12-week treatment, similar to the other psychiatric disorders examined in our study.

Previous studies have consistently demonstrated significant impairments in immediate and delayed memory in both SZ and BD II [41–44]. In this study, we conducted a correlation analysis between RBANS immediate and delayed memory scores and clinical improvements (PANSS scores for SZ and HAMD scores for BD II). The results revealed a significant positive correlation between immediate memory (learning and story memory) scores and PANSS scores for SZ, as well as between immediate memory (learning) and delayed memory (story recall) scores and HAMD scores for BD II. Impairments in immediate and delayed memory have been shown to impact the prognosis of SZ and BD II patients [45] and may contribute to the poor prognosis often observed in these disorders.

To date, antipsychotics have shown limited efficacy in improving cognition, and other pharmacological approaches for treating cognitive deficits have not shown strong effectiveness [30, 46]. The severity of psychiatric disorders may exacerbate cognitive symptoms [10]. Furthermore, cognitive symptoms are closely related to functional outcomes and contribute to the burden of the illness. Therefore, it is reasonable to believe that treatment for recently diagnosed drug-naïve psychiatric disorders can greatly enhance cognitive function.

However, several limitations should be acknowledged in our study. Firstly, this study was observational in nature, and the treatment approach was tailored to each patient's condition, which may introduce the influence of drug-related factors on the results. Secondly, our study only included BD II, as BD I pose a higher risk of impulsivity and often exhibits lower cooperation rates, resulting in a high dropout rate. Therefore, the cognitive evaluation results may not be generalizable to BD I.

Finally, we did not assess depression scores for the SZ group, and depressive symptoms in SZ have been shown to be related to cognition and functioning.

## Conclusion

In conclusion, our study provides evidence that patients with BD II and SZ share similar patterns of cognitive deficits across different domains. Standardized clinical treatment plays a crucial role in improving cognitive function, specifically immediate and delayed memory, in patients with BD II and SZ. However, further research is needed to investigate cognitive function differences among BD II, SZ, and MDD patients during periods of remission, utilizing larger sample sizes, highly consistent methodologies, and long-term follow-up.

## Data Availability

Data is provided within the manuscript.
